# Morphological and phylogenetic characterisation of novel *Cytospora* species associated with mangroves

**DOI:** 10.3897/mycokeys.38.28011

**Published:** 2018-08-28

**Authors:** Chada Norphanphoun, Olivier Raspé, Rajesh Jeewon, Ting-Chi Wen, Kevin D. Hyde

**Affiliations:** 1 The Engineering Research Center of Southwest Bio-Pharmaceutical Resources, Ministry of Education, Guizhou University, Guiyang, 550025, China Mae Fah Luang University Chiang Rai Thailand; 2 Center of Excellence in Fungal Research, Mae Fah Luang University, Chiang Rai, 57100 Thailand Guizhou University Guiyang China; 3 Botanic Garden Meise Nieuwelaan, 38, 1860, Meise, Belgium Botanic Garden Meise Nieuwelaan Meise Belgium; 4 Fédération Wallonie-Bruxelles, Service général de l’Enseignement supérieur et de la Recherche scientifique, rue A. Lavallée 1, 1080 Brussels, Belgium Fédération Wallonie-Bruxelles Brussels Belgium; 5 Department of Health Sciences, Faculty of Science, University of Mauritius, Reduit, 80837, Mauritius University of Mauritius Moka Mauritius

**Keywords:** 3 new species, Cytosporaceae, *
Lumnitzera racemosa
*, Mangroves, Phylogeny, Taxonomy, *
Xylocarpus granatum
*, *
Xylocarpus moluccensis
*

## Abstract

Mangroves are relatively unexplored habitats and have been shown to harbour a number of novel species of fungi. In this study, samples of microfungi were collected from symptomatic branches, stem and leaves of the mangrove species *Xylocarpusgranatum*, *X.moluccensis* and *Lumnitzeraracemosa* and examined morphologically. The phylogeny recovered supports our morphological data to introduce three new species, *Cytosporalumnitzericola*, *C.thailandica* and *C.xylocarpi*. In addition, a combined multi-gene DNA sequence dataset (ITS, LSU, ACT and RPB2) was analysed to investigate phylogenetic relationships of isolates and help in a more reliable species identification.

## Introduction

Mangroves are forests established in tropical and subtropical backwaters, estuaries, deltas and lagoons. These forests play a major role in the ecology of coastal tropical/subtropical waters, as they serve as hatchery and nursery habitats for marine organisms and protect coastlines from catastrophic events such as storms and tidal surges (Hyde and Jones 1988, Fisher and Spalding 1993, Hyde and Lee 1995, Hyde et al. 1998). The greatest diversity of mangrove species occurs in the mangroves of Indonesia, Malaysia and Thailand (Alias and Jones 2009, Alias et al. 2010).

Reports of fungi associated with mangroves are relatively few and data on diseases of mangroves are uncommon (Cribb and Cribb 1955, Kohlmeyer and Kohlmeyer 1979, Hyde and Jones 1988). So far, a number of fungi collected from mangroves are either saprobes (e.g Swe et al. 2008a, b, Devadatha et al. 2018, Li et al. 2018) or endophytes (e.g Liu et al. 2012, Doilom et al. 2017). One early species documented from mangroves is that of Stevens (1920) who reported a species of *Anthostomella* that was found from a leaf spot in red mangroves (*Rhizophoramangle*) in Puerto Rico. Later, McMillan (1964) reported *Cercospora* which caused leaf spot on red mangroves in Florida and Kohlmeyer (1969) documented an undescribed *Cytospora* species on *R.mangle* in Hawaii. *Cytosporarhizophorae* has also been reported as a marine fungus from *Rhizophoramangle* in southwest Puerto Rico (Wier et al. 2000). Later, Shivas et al. (2009) reported a serious disease, caused by *Pseudocercosporaavicenniae*, on leaves of *Avicenniamarina* in Cape Tribulation, Queensland.

*Cytospora* was introduced by Ehrenberg (1818) and belongs to the family Cytosporaceae in *Diaporthales* (Wijayawardene et al. 2018). *Cytospora* species are phytopathogens or saprobes (Wehmeyer 1975, Barr 1978, Eriksson 2001, Castlebury et al. 2002, Wijayawardene et al. 2018). *Cytospora* has a worldwide distribution and is an important pathogenic genus, causing canker and dieback disease on branches of a wide range of plants (Adams et al. 2005, 2006, Hyde et al. 2017, Norphanphoun et al. 2017). Currently, there are 614 epithets for *Cytospora* (Index Fungorum 2018, 14 June 2018) with an estimated 110 species in Kirk et al. (2008). Recently, fourteen new species were introduced to this genus by Norphanphoun et al. (2017). In this study, we report on three novel species of *Cytospora* associated with mangroves in Thailand. Detailed descriptions and illustrations of all the species identified are provided in this paper.

## Material and methods

### Sample collection and examination of specimens

Samples collected were dead branches of *Xylocarpusgranatum* K.D. Koenig, *X.moluccensis* (Lam.) M. Roem. and leaf spots of *Lumnitzeraracemosa* Willd. from Phetchaburi and Ranong provinces, Thailand in 2016. Specimens were returned to the laboratory in paper bags, examined and described following Norphanphoun et al. (2017). Morphological characters of ascomata and conidiomata were examined using a Motic SMZ 168 dissecting microscope. Hand sections were mounted in water and examined for morphological details. Micro-morphology was studied using a Nikon Ni compound microscope and photographed with a Canon EOS 600D digital camera fitted to the microscope. Photo-plates were made using Adobe Photoshop CS6 Extended version 13.0 × 64 (Adobe Systems, USA), while Tarosoft (R) Image Frame Work programme v. 0.9.7 was used for measurements.

Cultures were obtained by single spore isolation method outlined in Chomnunti et al. (2014). Single germinating spores were observed and photographed using a Nikon Ni compound microscope fitted with Canon EOS 600D digital camera. Geminated spores were transferred aseptically to 2% malt extract agar (MEA, malt extract agar powder 32 g in 1000 ml water) and incubated at room temperature (18−25 °C). A tissue isolation method was used for isolation of taxa from leaf spots of *Lumnitzeraracemosa*. Leaves with leaf spots were cut into small pieces (0.5 × 0.5 cm^2^) using a sterilised blade and surface was sterilised using 70% ethanol for 1 minute, followed by three rinses with sterile distilled water, 1 minute in 3% sodium hypochlorite (NaOCl) and rinsed with sterile water for 1–2 minutes and dried by blotting on sterile filter paper. Four to five segments including the edge of the leaf spot were placed on water agar (WA) plates, supplemented with 100 mg/ml streptomycin. The dishes were incubated at 27 °C ± 2 °C for 7–10 days. Fungal colonies were transferred using single hyphal tips on to potato dextrose agar (PDA) plates throughout a 2-week period. Pure cultures were maintained for further studies on PDA (Bharathidasan and Panneerselvam 2011). The specimens/dried cultures and living cultures are deposited in the Herbarium Mae Fah Luang University (MFLU) and culture collection Mae Fah Luang University (MFLUCC), Chiang Rai, Thailand and duplicated in the International Collection of Micro-organisms from Plants (ICMP). Facesoffungi numbers were registered as in Jayasiri et al. (2015). New taxa are established based on recommendations as outlined by Jeewon and Hyde (2016).

### DNA extraction, PCR amplification and sequencing

Genomic DNA was extracted from fresh fungal mycelia growing on MEA at room temperature (18−25 °C) for three weeks using a E.Z.N.A.TM Fungal DNA MiniKit (Omega Biotech, CA, USA) following the manufacturer’s protocols. Polymerase chain reactions (PCR) were carried out using primer pairs of ITS1 (5'-TCCGTAGGTGAACCTGCGG-3') and ITS4 (5'-TCCTCCGCTTATTGATATGC-3') to amplify the ITS region (White et al. 1990), primer pairs of NL1 (5'-GCATATCAATAAGCGGAGGAAAAG-3') and NL4 (5'-GGTCCGTGTTTCAAGACGG-3') to amplify part of the large subunit rDNA (28S, LSU) (O’Donnell 1993), the partial ACT region was amplified using primers ACT512F (5'-ATGTGCAAGGCCGGTTTCGC-3') and ACT783R (5'-TACGAGTCCTTCTGGCCCAT-3') (Carbone and Kohn 1999) and the partial RPB2 region was amplified using primers bRPB2-6F (5'-TGGGGYATGGTNTGYCCYGC-3') and bRPB2-7.1R (5'-CCCATRGCYTGYTTMCCCATDGC-3') (Matheny 2005).

The amplification reactions were carried out with the following protocol: 50 μl reaction volume containing 2 µl of DNA template, 2 µl of each forward and reverse primers, 25 µl of 2 × Bench Top^TM^Taq Master Mix (mixture of Taq DNA Polymerase (recombinant): 0.05 units/µl, MgCl_2_: 4 mM and dNTPs (dATP, dCTP, dGTP, dTTP): 0.4 mM) and 19 µl of double-distilled water (ddH_2_O) (sterilised water) using the thermal cycle programme in Norphanphoun et al. (2017). Purification and sequencing of PCR products with the same primers mentioned above were carried out at Life Biotechnology Co., Shanghai, China.

### Phylogenetic analysis

The sequences were assembled by GENEIOUS Pro v. 11.0.5 (Biomatters) and BLAST searches were made to retrieve the closest matches in GenBank and multiple alignment also included recently published sequences (Norphanphoun et al. 2017, Hyde et al. 2017, 2018). Combined analyses of ITS1, 5.8S, ITS2, LSU, RPB2 and ACT sequence data of 86 taxa were performed under different optimality criteria (MP, ML, BI). *Diaportheeres* (AFTOL-ID 935) was used as the outgroup taxon. In order to obtain a better picture of the phylogenetic relationships amongst our strains and closely related strains, a separate ITS1+ITS2 phylogeny was inferred, because only ITS sequences were available for many strains in that group and because less ambiguously aligned (and excluded) positions are expected in a dataset with narrower taxonomic coverage. Nineteen strains were selected for this analysis based on preliminary analyses and results from the multigene phylogeny. All sequences were aligned separately using the MAFFT v.7.110 online programme (http://mafft.cbrc.jp/alignment/server/; Katoh and Standley 2013) and Gblocks v. 0.91b was used to exclude ambiguously aligned positions in the ITS and ACT alignments (Castresana 2000, Talavera and Castresana 2007). A partition homogeneity test (PHT) was performed with PAUP 4.0b10* (Swofford 2002) to determine whether the individual datasets were congruent and could be combined. The combined sequence alignments were obtained from MEGA7 version 7.0.14 (Kumar et al. 2015), missing data were coded as question marks (?) and further manual adjustments were made wherever necessary in BioEdit 7.2.3 (Hall 1999). The combined sequence alignment was converted to NEXUS file for maximum parsimony analysis using ClustalX v. 2 (Larkin et al. 2007). The NEXUS file was prepared for MrModeltest v. 2.2 (Nylander 2004) in PAUP v.4.0b10 (Swofford 2002).

Maximum Parsimony (MP) analysis was performed using PAUP (Phylogenetic Analysis Using Parsimony) v. 4.0b10* (Swofford 2002) with 1000 bootstrap replicates using a heuristic search with random stepwise addition and tree-bisection reconnection (TBR), as detailed by Jeewon et al. (2002) and Cai et al. (2005). Maxtrees was set to 1000, branches of zero length were collapsed. The following descriptive tree statistics were calculated: parsimony tree length [TL], consistency index [CI], retention index [RI], rescaled consistency index [RC] and homoplasy index [HI].

For both Maximum Likelihood and Bayesian analyses, a partitioned analysis was performed with the following six partitions: ITS1+ITS2, 5.8S, LSU, ACT-exons, ACT-introns and RPB2. Maximum-likelihood (ML) analysis was performed with RAxML (Stamatakis 2006) implemented in the CIPRES Science Gateway web server (RAxML-HPC2 on XSEDE; Miller et al. 2010), 25 categories, 1000 rapid bootstrap replicates were run with the GTRGAMMA model of nucleotide evolution. Maximum likelihood bootstrap values (MLBS) equal or greater than 50% are given above each node.

Bayesian Inference (BI) analysis was performed using the Markov Chain Monte Carlo (MCMC) method with MrBayes 3.2.2 (Ronquist et al. 2012). The best-fit nucleotide substitution model for each dataset was separately determined using MrModeltest version 2.2 (Nylander 2004). GTR+I+G was selected as the best-fit model for the ITS1+ITS2, LSU, ACT (ACT-exons and ACT-introns) and RPB2 datasets and K80 for 5.8S. The MCMC analyses, with four chains starting from random tree topology, were run for 5,000,000 or 10,000,000 generations for the combined dataset or the ITS1+ITS2 dataset. Trees were sampled every 100 generations. Tracer v. 1.5.0 was used to check the effective sampling sizes (ESS) that should be above 200, the stable likelihood plateaus and burn-in value (Rambaut et al. 2013). The first 5000 samples were excluded as burn-in.

The phylogram was visualised in FigTree v1.4.0 (http://tree.bio.ed.ac.uk/software/figtree/; Rambaut 2014) and edited in Adobe Illustrator CC and Adobe Photoshop CS6 Extended version 13.1.2 × 64. Newly generated sequences in this study are deposited in GenBank. The finalised alignment and tree were deposited in TreeBASE, submission ID: 22942 (combined sequence alignment) (Reviewer access URL: http://purl.org/phylo/treebase/phylows/study/TB2:S22942?x-access-code=f9115cf637b0e4171aab1c980eb15830&format=html) and (Reviewer access URL: http://purl.org/phylo/treebase/phylows/study/TB2:S22943?x-access-code=92a782825ac069b3fd761aff21fa2bf4&format=html) 22943 (ITS sequence alignment) (http://www.treebase.org).

**Table 1.** GenBank accession numbers of the sequences used in phylogenetic analyses.

No	Taxon	Strain^a^	Host	Origin	GenBank accession numbers				References
					ITS	LSU	RPB2	ACT	
1	* Cytospora abyssinica *	CMW 10181^T^	* Eucalyptus globulus *	Wondo Genet, Ethiopia	AY347353	–	–	–	Adams et al. (2005)
2	* C. acaciae *	CBS 468.69	* Ceratonia siliqua *	Spain, Mallorca	DQ243804	–	–	–	Adams et al. (2006)
3	* C. ampulliformis *	MFLUCC 16-0583^T^	* Sorbus intermedia *	Russia	KY417726	KY417760	KY417794	KY417692	Norphanphoun et al. (2017)
4	* C. atrocirrhata *	HMBF156			KF225610	KF225624	–	KF498673	Fan et al. (2015a)
5	* C. austromontana *	CMW 6735^T^	* Eucalyptus pauciflora *	Australia	AY347361	–	–	–	Adams et al. (2005)
6	* C. berberidis *	CFCC 89927^T^	* Berberis dasystachya *	China	KR045620	KR045702	KU710948	KU710990	Liu et al. (2015)
7	* C. berkeleyi *	StanfordT3^T^	* Eucalyptus globulus *	California, USA	AY347350	–	–	–	Adams et al. (2005)
8	* C. brevispora *	CBS 116829	* Eucalyptus grandis *	Venezuela	AF192321	–	–	–	Adams et al. (2005)
9	* C. carbonacea *	CFCC 89947	* Ulmus pumila *	Qinghai, China	KR045622	KP310812	KU710950	KP310842	Yang et al. (2015)
10	* C. centravillosa *	MFLUCC 16-1206^T^	* Sorbus domestica *	Italy	MF190122	MF190068	MF377600	–	Senanayake et al. (2017)
11	* C. ceratosperma *	MFLUCC 16-0625	* Acer platanoides *	Russia	KY563246	KY563248	KY563244	KY563242	Tibpromma et al. (2017)
12	* C. chrysosperma *	HMBF151			KF225605	KF225619	–	KF498668	Fan et al. (2015a)
13	* C. cinereostroma *	CMW 5700^T^	* Eucalyptus globulus *	Chile	AY347377	–	–	–	Adams et al. (2005)
14	* C. cotini *	MFLUCC 14-1050^T^	* Cotinus coggygria *	Russia	KX430142	KX430143	KX430144	–	Norphanphoun et al. (2017)
15	* C. curvata *	MFLUCC 15-0865 ^T^	* Salix alba *	Russia	KY417728	–	–	KY417694	Norphanphoun et al. (2017)
16	* C. cypri *	CBS 201.42^T^	*Syringa* sp.	Switzerland	DQ243801	–	–	–	Adams et al. (2006)
17	* C. diatrypelloidea *	CMW 8549^T^	* Eucalyptus globulus *	Orbost, Australia	AY347368	–	–	–	Adams et al. (2005)
18	* C. disciformis *	CMW6509			AY347374	–	–	–	Adams et al. (2005)
19	* C. donetzica *	MFLUCC 16-0574^T^	*Rosa* sp.	Russia	KY417731	KY417765	KY417799	KY417697	Norphanphoun et al. (2017)
20	* C. elaeagni *	CFCC 89632	* Elaeagnus angustifolia *	Ningxia, China	KR045626	KR045706	KU710955	KU710995	Fan et al. (2015b)
21	* C. erumpens *	MFLUCC 16-0580^T^	Salix × fragilis	Russia	KY417733	KY417767	KY417801	KY417699	Norphanphoun et al. (2017)
22	* C. eriobotryae *	IMI136523 ^T^	* Eriobotrya japonica *	India	AY347327	–	–	–	Adams et al. (2005)
23	* C. eucalypti *	LSEQ	* Sequoia sempervirens *	California, USA	AY347340	–	–	–	Adams et al. (2005)
24	* C. eucalyptina *	CMW 5882	* Eucalyptus grandis *	Cali, Columbia	AY347375	–	–	–	Adams et al. (2005)
25	* C. fabianae *	Dunnii	* Eucalyptus *		AY347360	–	–	–	Adams et al. (2005)
26	* C. friesii *	CBS 113.81	* Picea abies *	Norway	AY347318	–	–	–	Adams et al. (2005)
27	* C. gelida *	MFLUCC 16-0634 ^T^	* Cotinus coggygria *	Russia	KY563245	KY563247	KY563243	KY563241	Tibpromma et al. (2017)
28	* C. germanica *	CXY1322	* Elaeagnus oxycarpa *	China	JQ086563	JX524617	–	–	Zhang et al. (2013)
29	* C. gigalocus *	HMBF154			KF225608	KF225622	–	KF498671	Fan et al. (2015a)
30	* C. gigaspora *	CFCC 89634^T^	* Salix psammophila *	China	KF765671	KF765687	KU710960	KU711000	Fan et al. (2015b)
31	* C. hippophaes *	CFCC 89636			KF76567878	KF765694	KF765710	–	Fan et al. (2015b)
32	* C. japonica *	CBS375.29	* Prunus persica *	Japan	AF191185	–	–	–	Adams et al. (2002)
33	* C. junipericola *	MFLUCC 17-0882^T^	* Juniperus communis *	Italy	MF190125	MF190072	–	–	Senanayake et al. (2017)
34	* C. kantschavelii *	287-2	* Populus deltoides *	Iran	EF447367	–	–	–	Fotouhifar et al. (2010)
35	* C. kunzei *	CBS 118556	* Pinus radiata *	Eastern Cape, SA	DQ243791	–	–	–	Adams et al. (2006)
36	* C. leucostoma *	CFCC 50015	* Sorbus pohuashanensis *	China	KR045634	KR045714	–	KU711002	Yang et al. (2015)
37	* C. longiostiolata *	MFLUCC 16-0628^T^	Salix × fragilis	Russia	KY417734	KY417768	KY417802	KY417700	Norphanphoun et al. (2017)
38	* C. lumnitzericola *	MFLUCC 17-0508	* Lumnitzera racernosa *	Phetchaburi, Thailand	MG975778	MH253461	MH253453	MH253457	In this study
39	* C. mali *	CFCC 50044	* Malus baccata *	Haidong, Qinghai	KR045637	KR045717	KU710966	KU711005	Yang et al. (2015)
40	* C. malicola *	167			EF447414	–	–	–	Adams et al. (2002)
41	* C. mali-sylvestris *	MFLUCC 16-0638 ^T^	* Malus sylvestris *	Russia	KY885017	KY885018	KY885020	KY885019	Hyde et al. (2017)
42	* C. melnikii *	MFLUCC 15-0851^T^	* Malus domestica *	Russia	KY417735	KY417769	KY417803	KY417701	Norphanphoun et al. (2017)
43	* C. multicollis *	CBS 105.89^T^	Quercus ilex subsp. rotundifolia	Spain	DQ243803	–	–	–	Adams et al. (2006)
44	* C. myrtagena *	HiloTib1^T^	* Tibouchiina urvilleana *	Hilo, Hawaii	AY347363	–	–	–	Adams et al. (2005)
45	* C. nitschkii *	CMW10180^T^	* Eucalyptus globulus *	Wondo Genet, Ethiopia	AY347356	–	–	–	Adams et al. (2005)
46	* C. nivea *	MFLUCC 15-0860	* Salix acutifolia *	Russia	KY417737	KY417771	KY417805	KY417703	Norphanphoun et al. (2017)
47	* C. palmae *	CXY1280^T^	* Cotinus coggygria *	Beijing, China	JN411939	–	–	–	Zhang et al. (2014)
48	* C. parakantschavelii *	MFLUCC 15-0857^T^	Populus × sibirica	Russia	KY417738	KY417772	KY417806	KY417704	Norphanphoun et al. (2017)
49	* C. parapersoonii *	T28.1^T^	* Prunus persicae *	Michigan, USA	AF191181	–	–	–	Adams et al. (2002)
50	* C. paratranslucens *	MFLUCC 16-0506^T^	Populus alba var. bolleana	Russia	KY417741	KY417775	KY417809	KY417707	Norphanphoun et al. (2017)
51	* C. parasitica *	MFLUCC 16-0507	* Malus domestica *	Russia	KY417740	KY417774	KY417808	KY417706	Norphanphoun et al. (2017)
52	* C. pini *	CBS224.52^T^	* Pinus strobus *	New York	AY347316	–	–	–	Adams (2005)
53	* C. populina *	CFCC 89644	* Salix psammophila *	Shaanxi, China	KF765686	KF765702	KF765718	–	Fan et al. (2015b)
54	* C. predappioensis *	MFLU 17-0327	* Platanus hybrida *	Italy	MH253451	MH253452	MH253450	MH253449	Hyde et al. (2018)
55	* C. prunicola *	MFLU 17-0995 ^T^	*Prunus* sp.	Italy	MG742350	MG742351	MG742352	MG742353	Hyde et al. (2018)
56	* C. pruinopsis *	CFCC 50034^T^	* Ulmus pumila *	Shaanxi, China	KP281259	KP310806	KU710970	KP310836	Yang et al. (2015)
57	* C. pruinosa *	CFCC 50036	* Syzygium aromaticum *	Qinghai, China	KP310800	KP310802	–	KP310832	Yang et al. (2015)
58	* C. quercicola *	MFLUCC 14-0867^T^	*Quercus* sp.	Italy	MF190129	MF190073	–	–	Senanayake et al. (2017)
59	* C. rhizophorae *	ATCC38475	* Rhizophora mangle *	LA, USA	DQ996040	–	–	–	He et al. (2003)
60	* C. rhizophorae *	ATCC66924	* Haliclona caerulea *	HI, USA	DQ092502	–	–	–	Unpublished
61	* C. ribis *	CFCC 50026	* Ulmus pumila *	Qinghai, China	KP281267	KP310813	KU710972	KP310843	Yang et al. (2015)
62	* C. rosae *	MFLUCC 14-0845^T^	* Rosa canina *	Italy	MF190131	MF190075	–	–	Senanayake et al. (2017)
63	* C. rosarum *	218			EF447387	–	–	–	Fotouhifar et al. (2010)
64	* C. rostrata *	CFCC 89909^T^	* Salix cupularis *	Gansu, China	KR045643	KR045722	KU710974	KU711009	Unpublished
65	* C. rusanovii *	MFLUCC 15-0854^T^	* Salix babylonica *	Russia	KY417744	KY417778	KY417812	KY417710	Norphanphoun et al. (2017)
66	* C. sacculus *	HMBF281			KF225615	KF225629	–	KF498678	Fan et al. (2015a)
67	* C. salicacearum *	MFLUCC 16-0509^T^	* Salix alba *	Russia	KY417746	KY417780	KY417814	KY417712	Norphanphoun et al. (2017)
68	* C. salicicola *	MFLUCC 14-1052^T^	* Salix alba *	Russia	KU982636	KU982635	–	KU982637	Li et al. (2016)
69	* C. salicina *	MFLUCC 15-0862^T^	* Salix alba *	Russia	KY417750	KY417784	KY417818	KY417716	Norphanphoun et al. (2017)
70	* C. schulzeri *	CFCC 50040	* Malus domestica *	Ningxia, China	KR045649	KR045728	KU710980	KU711013	Unpublished
71	* C. sibiraeae *	CFCC 50045^T^	* Sibiraea angustata *	Gansu, China	KR045651	KR045730	KU710982	KU711015	Liu et al. (2015)
72	* C. sorbi *	MFLUCC 16-0631^T^	* Sorbus aucuparia *	Russia	KY417752	KY417786	KY417820	KY417718	Norphanphoun et al. (2017)
73	* C. sorbicola *	MFLUCC 16-0584^T^	* Acer pseudoplatanus *	Russia	KY417755	KY417789	KY417823	KY417721	Norphanphoun et al. (2017)
74	* C. sordida *	HMBF159			KF225613	KF225627	–	KF498676	Fan et al. (2015a)
75	* C. sophorae *	CFCC 50047	* Styphnolobium japonicum *	Shanxi, China	KR045653	KR045732	KU710984	KU711017	Fan et al. (2014)
76	* C. sophoricola *	CFCC 89596	* Styphnolobium japonicum *	Gansu, China	KR045656	KR045735	KU710987	KU711020	Unpublished
77	* C. tanaitica *	MFLUCC 14-1057^T^	* Betula pubescens *	Russia	KT459411	KT459412	–	KT459413	Ariyawansa et al. (2015)
78	* C. thailandica *	MFLUCC 17-0262	* Xylocarpus moluccensis *	Ranong, Thailand	MG975776	MH253463	MH253455	MH253459	In this study
79	* C. thailandica *	MFLUCC 17-0263	* Xylocarpus moluccensis *	Ranong, Thailand	MG975777	MH253464	MH253456	MH253460	In this study
80	* C. tibouchinae *	CPC 26333^T^	* Tibouchina semidecandra *	La Reunion, France	KX228284	KX228335	–	–	Unpublished
81	* C. translucens *	35			EF447403	–	–	–	Fotouhifar et al. (2010)
82	* C. ulmi *	MFLUCC 15-0863^T^	* Ulmus minor *	Russia	KY417759	KY417793	KY417827	KY417725	Norphanphoun et al. (2017)
83	* C. valsoidea *	CMW 4309^T^	* Eucalyptus grandis *	Sibisa, North Sumatra	AF192312	–	–	–	Adams et al. (2005)
84	* C. variostromatica *	CMW 6766^T^	* Eucalyptus globulus *	Australia	AY347366	–	–	–	Adams et al. (2005)
85	* C. vinacea *	CBS 141585^T^	*Vitis* sp.	New Hampshire, USA	KX256256	–	–	–	Lawrence et al. (2017)
86	* C. xylocarpi *	MFLUCC 17-0251	* Xylocarpus granatum *	Ranong, Thailand	MG975775	MH253462	MH253454	MH253458	In this study
87	* Diaporthe eres *	AFTOL-ID 935			DQ491514	–	DQ470919	–	Spatafora et al. (2006)
88	*C.* “rhizophorae”	A761	* Morinda officinalis *	China	KU529867	–	–	–	Unpublished
89	*C.* “rhizophorae”	HAB16R13	* Cinnamomum porrectum *	Malaysia	HQ336045	–	–	–	Harun et al. (2011)
90	*C.* “rhizophorae”	M225	* Rhizophora mucronata *	Philippines	KR056292	–	–	–	Unpublished
91	*C.* “rhizophorae”	MUCC302	* Eucalyptus grandis *	Australia	EU301057	–	–	–	Unpublished

^a^*AFTOL-ID* Assembling the Fungal Tree of Life; *CBS* CBS-KNAW Fungal Biodiversity Centre, Utrecht, The Netherlands; *CFCC* China Forestry Culture Collection Center; IMI International Mycological Institute, CABI-Bioscience, Egham, Bakeham Lane, UK; *CPC* Culture collection of Pedro Crous, housed at CBS; *MFLU* Mae Fah Luang University Herbarium Collection; *MFLUCC* Mae Fah Luang University Culture Collection, Chiang Rai, Thailand; ^T^ Ex-type and ex-epitype cultures.

## Results

### Phylogenetic analysis of combined ITS, LSU, ACT and RPB2 sequences

**Figure 1.**
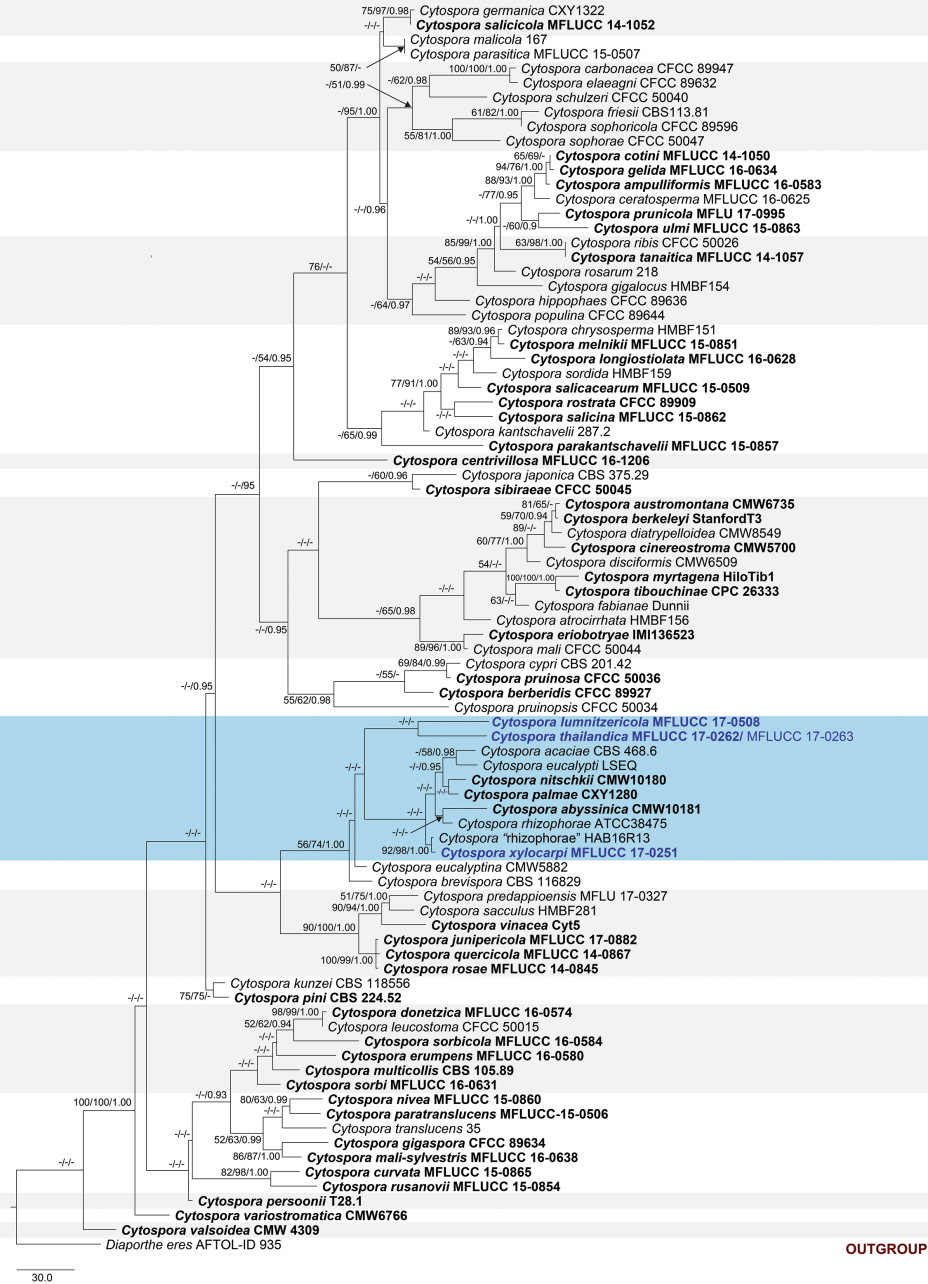
Phylogram generated from maximum parsimony analyses based on analysis of combined ITS, LSU, ACT and RPB2 sequence data. The tree is rooted to *Diaportheeres* (AFTOL-ID 935). Maximum parsimony and maximum likelihood bootstrap values ≥50%, Bayesian posterior probabilities ≥0.90 (MPBS/MLBS/PP) are given at the nodes. The species obtained in this study are in blue font. Ex-type taxa from other studies are in black bold.

**Figure 2.**
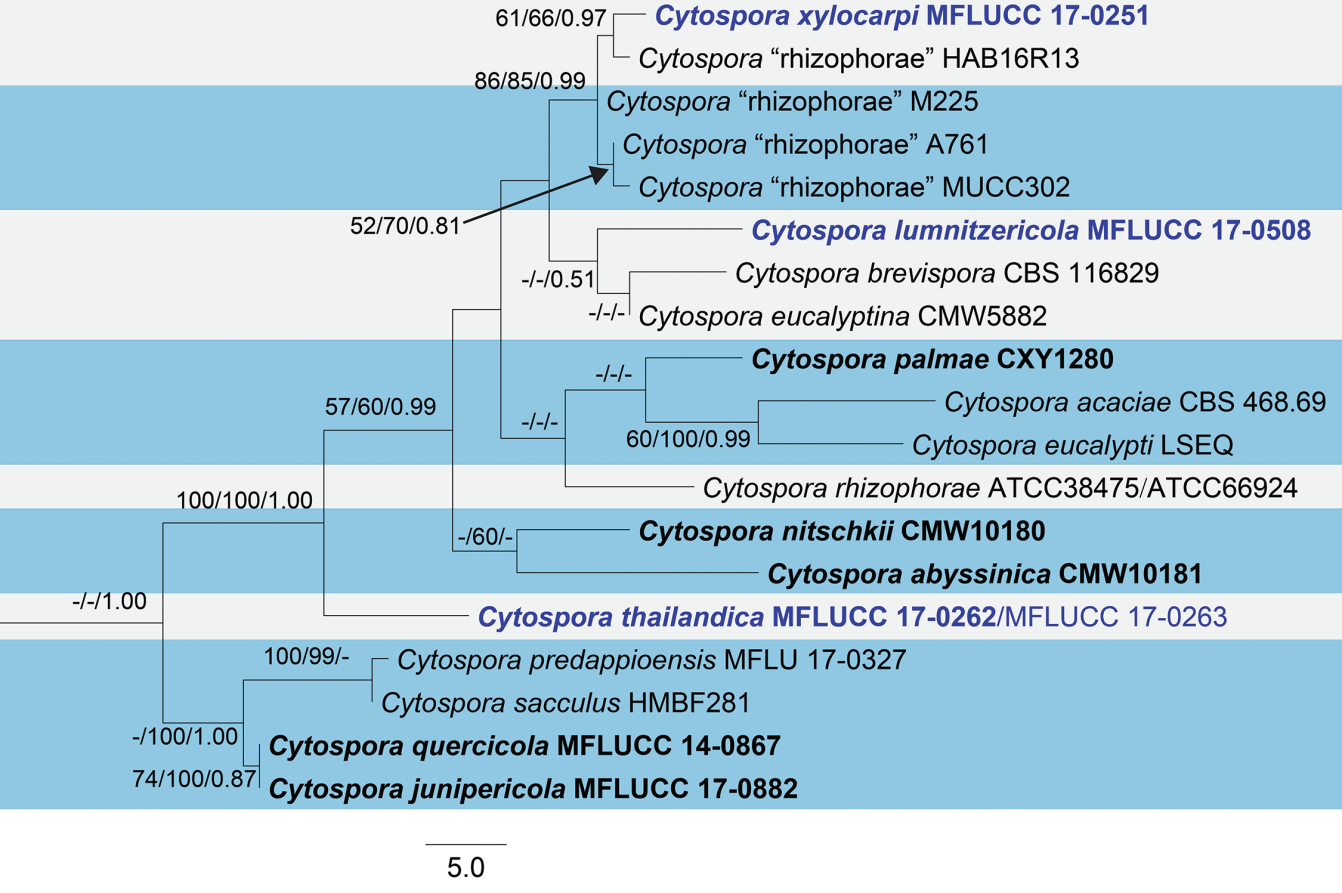
Maximum parsimony phylogenetic tree inferred from ITS1 and ITS2 sequence data. Maximum parsimony and maximum likelihood bootstrap values ≥50%, Bayesian posterior probabilities ≥0.90 (MPBS/MLBS/BIPP) are given at the nodes. The species obtained in this study are in blue font. Ex-type taxa from other studies are in black bold.

The combined alignment of ITS, LSU, ACT and RPB2 sequences comprised 86 taxa, including our strains, with *Diaportheeres* (CBS 183.5) as the outgroup taxon. The total length of the dataset was 2037 characters including alignment gaps (1–199, 200–357, 358–518, 519–1056, 1057–1296 and 1297–2037 corresponding to ITS1, 5.8S, ITS2, LSU, ACT and RPB2, respectively). The combined dataset contained 1426 constant, 144 parsimony uninformative and 467 parsimony informative characters. The result from the partition homogeneity test (PHT) was not significant (level 95%), indicating that the individual datasets were congruent and could be combined. The combined dataset was analysed using MP, ML and Bayesian analyses. The trees generated under different optimality criteria were essentially similar in topology and did not differ significantly (data not shown). The descriptive statistics of the phylogram generated from MP analysis based on the combined dataset of ITS, LSU, ACT and RPB2 (Fig. 1) were TL = 2418, CI = 0.375, RI = 0.650, RC = 0.244, HI = 0.625. The best scoring likelihood tree selected with a final value for the combined dataset = -14466.797686. The aligned sequence matrix of the ITS1+ITS2 dataset comprising 19 taxa had 279 constant, 23 parsimony uninformative and 57 parsimony informative characters. The descriptive statistics of the most parsimonious tree (Fig. 2) were TL = 2418, CI = 0.375, RI = 0.650, RC = 0.244, HI = 0.625. The best scoring likelihood tree obtained for the ITS1+ITS2 dataset had a log-likelihood of= -1276.782916.

### Taxonomy

#### Cytospora lumnitzericola

Taxon classificationFungiDiaporthalesValsaceae

Norphanphoun, T.C. Wen & K.D. Hyde sp. nov.

Figure 3

##### Etymology.

refers to the host where the fungus was isolated.

##### Holotype.

MFLU 18-1227

*Isolated from leaf spot* of *Lumnitzeraracemosa*. **Culture characteristic**: Colonies on MEA reaching 5–6 cm diameter after 2 days at room temperature, colonies circular to irregular, medium dense, flat or effuse, slightly raised, with edge fimbriate, fluffy to fairly fluffy, white to grey from above, light yellow to green from below; not producing pigments in agar. **Asexual morph**: *Conidiogenous cells* (8–)8.5–14 × 0.6–1.4(–1.6) μm (*x*‒ = 8.4 × 1.4, n = 15), blastic, enteroblastic, flask-shaped, phialidic, hyaline and smooth-walled. *Conidia* (3.7–)4–4.5 × 1–1.3(–1.5) µm (*x*‒ = 4 × 1.2 µm, n = 30), unicellular, subcylindrical, hyaline, smooth-walled.

##### Material examined.

THAILAND, Phetchaburi Province, the Sirindhorn International Environmental Park, on leaf spot of *Lumnitzeraracemosa*, 30 November 2016, Norphanphoun Chada NNS23-2a (MFLU 18-1227 dried culture, **holotype**; PDD, isotype); ex-type-living culture, MFLUCC 17-0508, ICMP.

##### Notes.

Based on the multigene phylogeny, *Cytosporalumnitzericola* is closely related to *Cytosporathailandica* (Fig. 1). Although conidial sizes of both species are similar, they have significant differences in nucleotides: ITS (26 nt), ACT (22 nt), and RPB2 (53 nt) (Table 5). The phylogeny derived from the ITS regions depicts *C.lumnitzericola* as an independent lineage close to *C.brevispora* CBS 116829 and *C.eucalyptina* CMW5882 (Fig. 2). In future, more collections are needed to confirm whether *C.lumnitzericola* can exist as a saprobe or endophyte as well as performing tests to confirm its pathogenicity.

**Figure 3.**
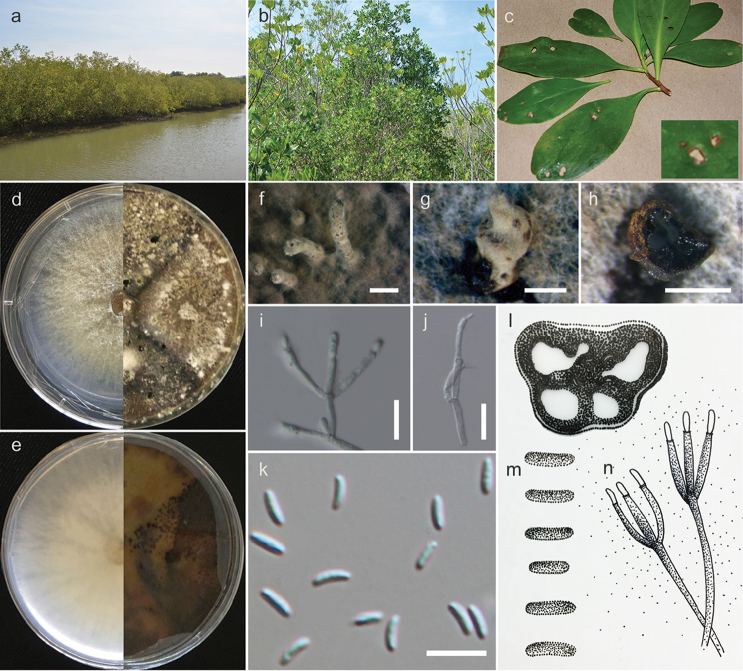
*Cytosporalumnitzericola* (MFLUCC 17-0508, from culture). **a** Mangrove collecting site **b, c***Lumnitzeraracemosa* in mangroves forest **d, e** Colonies on MEA after 6 days (left) and 30 days (right) (d-from above, e-from below) **f, g**Conidiomata produced on MEA**h, l** Transverse sections of conidioma **i, j, n** Conidiogenous cells with attached conidia **k, m**Conidia. Scale bars: **f** = 1000 µm, **g, h** = 500 µm, **i, j** = 10 µm, **k** = 5 µm.

#### Cytospora thailandica

Taxon classificationFungiDiaporthalesValsaceae

Norphanphoun, T.C. Wen & K.D. Hyde sp. nov.

Figure 4

##### Etymology.

refers to the country where the fungus was collected.

##### Holotype.

MFLU 17-0709

*Associated* with twigs and branches of *Xylocarpusmoluccensis*. **Sexual morph**: Stromata immersed in bark. *Ascostromata* 400–1000 × 70–250 µm diameter, semi-immersed in host tissue, scattered, erumpent, uni- or multi-loculate, with ostiolar neck. *Ostiole* 70–150 µm diameter, numerous, dark brown to black, at the same level as the disc, occasionally area below disc a lighter entostroma. *Peridium* comprising several layers of cell of *textura angularis*, with innermost layer thick, brown, outer layer dark brown. *Hamathecium* comprising long cylindrical, cellular, anastomosed paraphyses. *Asci* (21–)23–25 × 4.1–4.7(–5) μm (*x*‒ = 22 × 4.3 μm, n = 15), 6–8-spored, unitunicate, clavate to elongate obovoid, with a J-, refractive apical ring. *Ascospores* (5.6–)6–6.8 × 1.3–1.5(–2) μm (*x*‒ = 6.6 × 1.5 μm, n = 20), biseriate, elongate-allantoid, unicellular, hyaline, smooth-walled. **Asexual morph**: *Conidiomata* 400–1200 × 180–380 µm diameter, semi-immersed in host tissue, solitary, erumpent, scattered, discoid, circular to ovoid, with multi-loculate, pycnidial, embedded in stromatic tissue, with ostiole. *Ostioles* 230–300 µm long, with an ostiolar neck. *Peridium* comprising few layers of cells of *textura angularis*, with innermost layer thin, pale brown, outer layer brown to dark brown. *Conidiophores* unbranched or occasionally branched at the bases, formed from the innermost layer of pycnidial wall, with conidiogenous cells. *Conidiogenous cells* (3.3–)6–9.1 × 1–1.3(–1.7) μm (*x*‒ = 6 × 1.3 μm, n = 15), blastic, enteroblastic, flask-shaped, phialidic, hyaline and smooth-walled. *Conidia* (3.3–)3.8–4 × 1–1.3(–1.5) µm (*x*‒ = 3.8 × 1.3 µm, n = 30), unicellular, subcylindrical, hyaline, smooth-walled.

##### Material examined.

THAILAND, Ranong Province, Ngao Mangrove Forest, on branches of *Xylocarpusmoluccensis*, 6 December 2016, Norphanphoun Chada NG02a (MFLU 17-0709, **holotype**; PDD, isotype); ex-type-living cultures, MFLUCC 17-0262, MFLUCC 17-0263, ICMP.

##### Notes.

*Cytosporathailandica* was collected from branches of *Xylocarpusmoluccensis*. The new species resembles some other *Cytospora* species, but is characterised by uni- or multi-loculate ascomata/conidiomata with unicellular, subcylindrical and hyaline spores in both morphs. *Cytospora* species associated with *Xylocarpusgranatum* is also reported in this study as *C.xylocarpi* (MFLUCC 17-0251, Fig. 5). *Cytosporaxylocarpi* is similar to *C.thailandica* in its conidiomata being multi-loculate and in the length of conidia in the asexual morph (*C.xylocarpi*: conidia 3 × 1.1 µm versus 3.8 × 1.3 µm in *C.thailandica*). However, *C.thailandica* differs from *C.xylocarpi* in having shorter ostiolar necks and larger asci and ascospores (Table 2). Phylogenetic analysis of our combined gene also reveals *C.thailandica* is closely related to *C.lumnitzericola* (Fig. 1), but there are nucleotide differences as mentioned in notes of *C.lumnitzericola*. The individual ITS1+ITS2 phylogenetic tree also indicates that *C.thailandica* is distinct with good support (Fig. 2).

**Figure 4.**
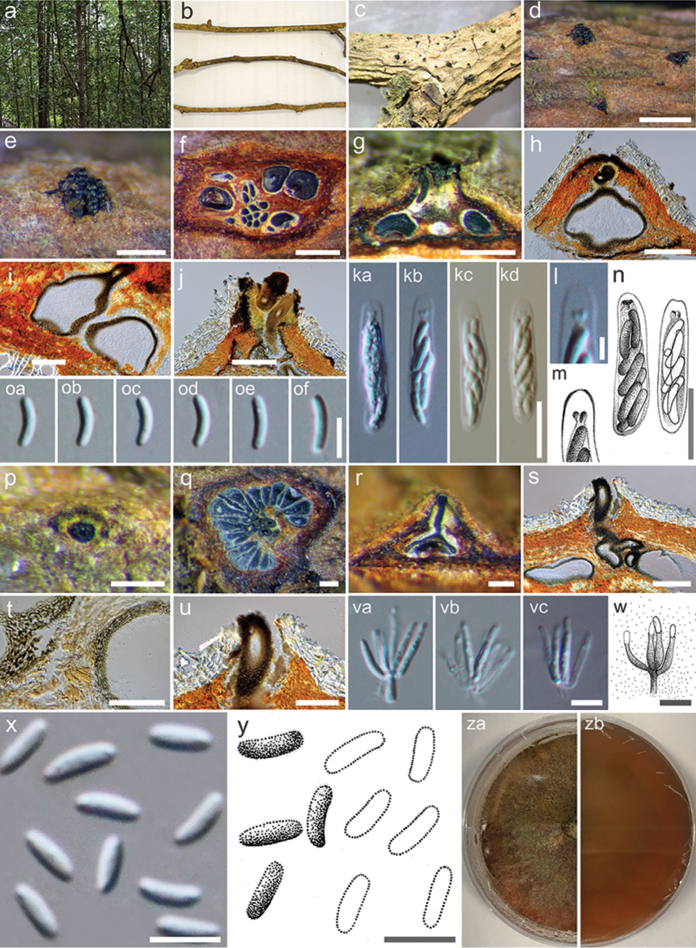
*Cytosporathailandica* (MFLU 17-0709, holotype). **a***Xylocarpusmoluccensis***b** Branch of *Xylocarpusmoluccensis***c**Ascostromata on host substrate **d, e** Surface of ascomata **f** Transverse sections through ascostroma to show distribution of locules **g–h** Longitudinal sections through ascostroma to show distribution of locules **i**Peridium**j** Ostiolar neck **ka**–**kd, n** Asci **l, m** Apical ring **oa–of**Ascospores**p** Surface of conidioma **q** Transverse sections through conidioma to show distribution of locules **r, s** Longitudinal sections through conidioma to show distribution of locules **t**Peridium**u** Ostiolar neck **va–vc, w** Conidiogenous cells with attached conidia **x, y**Conidia**za, zb** Colonies on MEA (za-from above, zb-from below). Scale bars: **d** = 1000 µm, **e–g** = 400 µm, **h, j, p–s** = 200 µm, **i, u** = 100 µm, **ka–kd, n** = 10 µm, **l, m** = 2 µm, **oa–of, va–vc, w** = 5 µm, **t** = 50 µm, **x, y** = 4 µm.

**Table 2.** Synopsis of species of *Cytospora* discussed in the paper.

Taxon	Sexual morph				Asexual morph				References
	Ascostoma	Ostiolar neck	Asci	Ascospores	Conidiomata	Ostiolar neck	Conidiogenous cell	Conidia	
* C. lumnitzericola *	–	–	–	–	–	–	8.4 × 1.4	4 × 1.2	In this study
* C. rhizophorae *	–	–	–	–	370–500 × 100–310	30 × 10–25	13–20 × 1–1.8	3–6 × 1.1–1.5	Kohlm. and Kohlm. (1971)
* C. thailandica *	400–1000 × 70–250	70–150	22 × 4.3	6.6 × 1.5	400–1200 × 180–380	230–300	6 × 1.3	3.8 × 1.3	In this study
* C. xylocarpi *	230–600 × 90–250	160–200	26 × 4	5.7 × 1.8	700–1200 × 400–480	200–250	8.5× 1.4	3 × 1	In this study

#### Cytospora xylocarpi

Taxon classificationFungiDiaporthalesValsaceae

Norphanphoun, T.C. Wen & K.D. Hyde sp. nov.

Figure 5

##### Etymology.

refers to the host genus that fungus was collected.

##### Holotype.

MFLU 17-0708

*Associated* with *Xylocarpusgranatum* branches. **Sexual morph**: Stromata immersed in bark. *Ascostromata* 230–600 × 90–250 µm diameter, semi-immersed in host tissue, scattered, erumpent, multi-loculate, with ostiolar neck. *Ostiole* 160–200 µm diameter, numerous, dark brown to black, at the same level as the disc, occasionally area surrounded with white hyphae. *Peridium* comprising several layers of cells of *textura angularis*, with innermost layer thick, pale brown, outer layer dark brown to black. *Hamathecium* comprising long cylindrical, cellular, anastomosed paraphyses. *Asci* (22–)24–28.8 × 3.6–4.8(–5.1) μm (*x*‒ = 26 × 4 μm, n = 15), 6–8-spored, unitunicate, clavate to elongate obovoid, with a refractive, J-, apical ring. *Ascospores* (5.5–)6–6.5 × 1.7–1.8(–2) μm (*x*‒ = 5.7 × 1.8 μm, n = 20), biseriate, elongate-allantoid, unicellular hyaline, smooth-walled. **Asexual morph**: *Conidiomata* 700–1200 × 400–480 µm diameter, semi-immersed in host tissue, solitary, erumpent, scattered, multi-loculate, with ostiole. *Ostioles* 200–250 µm long, with 1–2 ostiolar necks. *Peridium* comprising several layers of cells of *textura angularis*, with innermost layer brown, outer layer dark brown to black. *Conidiophores* unbranched or occasionally branched at the bases, formed from the innermost layer of pycnidial wall, with conidiogenous cells. *Conidiogenous cells* (6.3–)7.9–10 × 0.9–1.4(–1.6) μm (*x*‒ = 8.5× 1.4 μm, n = 15), blastic, enteroblastic, flask-shaped, phialidic, hyaline and smooth-walled. *Conidia* (2.4–)3–3.1 × 0.8–1(–1.2) µm (*x*‒ = 3 × 1 µm, n = 30), unicellular, subcylindrical, hyaline, smooth-walled.

##### Material examined.

THAILAND, Ranong Province, Ngao Mangrove Forest, on branches of *Xylocarpusgranatum*, 6 December 2016, Norphanphoun Chada NG09b (MFLU 17-0708, **holotype**; PDD); ex-type-living cultures, MFLUCC 17-0251, ICMP.

##### Notes.

The asexual morph of *C.xylocarpi*, studied here, is most similar to *C.rhizophorae* from dead roots of *Rhizophoramangle* L. in Guatemala, in having multi-loculate conidiomata and allantoid, slightly curved, hyaline and 3–6 × 1.1–1.5 μm conidia (Kohlmeyer and Kohlmeyer 1971). However, the phylogenies, generated herein, show that *C.xylocarpi* is distinct from *C.rhizophorae* (ATCC 38475), a strain from *Rhizophoramangle* that was identified by Kohlmeyer, the author of the species (Fig. 2). The two species also differ by 25 substitutions in ITS1+ITS2 and were collected from different hosts. Therefore, the collection in the present study is designated as a new species.

Our phylogeny also indicates a close relationship to unpublished sequences from GenBank (Figs 1, 2). Given that no morphological descriptions are available for these, the similarity in the ITS1 and ITS2 sequence between our strain and the sequences from GenBank (HAB16R13, M225, A761, MUCC302) are presented in Table 3. Those strains were collected from different hosts (Table 3) and, together with our strain, show substantial variation in ITS1 and ITS2 (Table 4). More collections are needed to further study morphological and genetic variation in this group.

**Figure 5.**
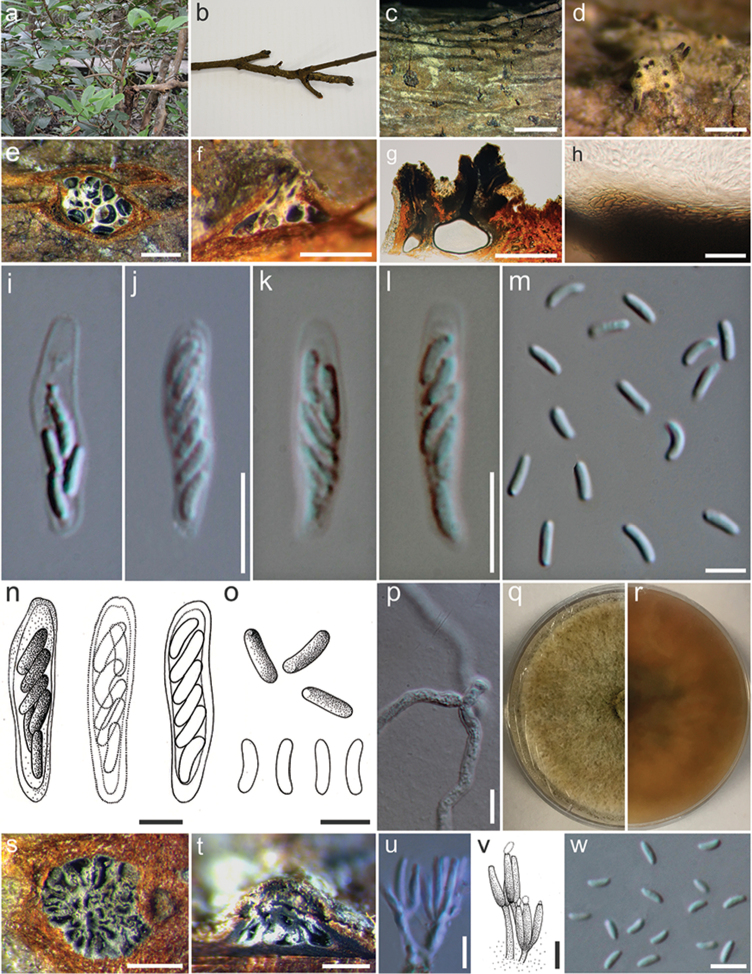
*Cytosporaxylocarpi* (MFLU 17-0708, holotype). **a***Xylocarpusgranatum***b** Branch of *Xylocarpusgranatum***c**Ascostromata on host substrate **d** Surface of ascomata **e** Transverse sections through ascostroma to show distribution of locules **f, g** Longitudinal sections through ascostroma to show distribution of locules **h**Peridium**i**–**l, n** Asci **m, o**Ascospores**p** Germinating spore **q, r** Colonies on MEA (q-from above, r-below) **s** Transverse sections through conidioma to show distribution of locules **t** Longitudinal sections through conidioma to show distribution of locules **u, v** Conidiogenous cells with attached conidia **w** Mature conidia. Scale bars: **c** = 2000 µm, **d–f** = 500 µm, **g** = 200 µm, **h** = 20 µm, **i, p** = 10 µm, **j–o, u–w** = 5 µm, **s, t** = 400 µm.

**Table 3.** GenBank BLAST search from ITS1 and ITS2 of *Cytosporaxylocarpi* (MFLUCC 17-0251) with sequence from GenBank identified as *Cytosporarhizophorae*.

Toxon	Strain	Host	Country	Accessions	ITS1	ITS2	ITS1+ITS2	Identities (I), Query cover (QC)	References
*C.* “rhizophorae”	HAB16R13	* Cinnamomum porrectum *	Malaysia	HQ336045	213/215	167/169	380/384	I=98.9%, QC=99%	Harun et al. (2011)
*C.* “rhizophorae”	M225	* Rhizophora mucronata *	Philippines	KR056292	213/217	167/169	380/386	I=98.4%, QC=100%	Unpublished
*C.* “rhizophorae”	A761	* Morinda officinalis *	China	KU529867	213/217	166/169	379/386	I=98.2%, QC=100%	Unpublished
*C.* “rhizophorae”	MUCC302	* Eucalyptus grandis *	Australia	EU301057	213/217	164/169	377/386	I=97.7%, QC=100%	Unpublished
* C. rhizophorae *	ATCC38475	* Rhizophora mangle *	LA, USA	DQ996040	187/202	156/166	343/368	I=93.2%, QC=100%	He et al. (2003)

**Table 4.** Nucleotide differences in the ITS1+ITS2 of *Cytosporaxylocarpi* (MFLUCC 17-0251) with sequence from GenBank identified as *Cytosporarhizophorae*.

Taxon	Strain	ITS1																	
		14	16	18	30	92	93	96	99	102	103	104	105	113	115	118	119	135	154
* C. xylocarpi *	MFLUCC 17-0251	-	G	A	C	C	C	C	G	G	G	C	G	C	T	T	C	A	G
* C. rhizophorae *	ATCC38475	G	A	C	T	G	A	T	A	T	T	T	A	T	-	C	T	-	T
*C.* “rhizophorae”	HAB16R13	?	G	A	C	C	C	C	G	G	G	C	G	C	T	T	C	A	T
*C.* “rhizophorae”	M225	?	G	A	T	C	C	C	G	G	G	C	G	C	T	T	C	A	T
*C.* “rhizophorae”	A761	?	G	A	T	C	C	C	G	G	G	C	G	C	T	T	C	A	T
*C.* “rhizophorae”	MUCC302	?	G	A	T	C	C	C	G	G	G	C	G	C	-	T	C	A	T
**Taxon**	**Strain**	**ITS2**																	
		**13**	**24**	**40**	**46**	**47**	**50**	**51**	**75**	**111**	**112**	**115**	**123**						
* C. xylocarpi *	MFLUCC 17-0251	C	C	A	T	-	T	T	C	A	A	C	T						
* C. rhizophorae *	ATCC38475	T	T	-	T	-	-	T	C	G	T	A	T						
*C.* “rhizophorae”	HAB16R13	C	T	A	T	-	T	T	C	A	A	C	C						
*C.* “rhizophorae”	M225	C	T	A	T	-	T	T	C	A	A	C	T						
*C.* “rhizophorae”	A761	C	T	A	T	T	-	T	C	A	A	C	T						
*C.* “rhizophorae”	MUCC302	C	T	A	-	-	-	-	T	A	A	C	T						

**Table 5.** Nucleotides differences in the ITS, ACT and RPB2 sequences of *Cytosporalumnitzericola*, *C.thailandica* and *C.xylocarpi*.

**Taxon**	**Strain**	**ITS**																	
		**29**	**88**	**91**	**92**	**93**	**94**	**96**	**97**	**99**	**101**	**102**	**103**	**104**	**105**	**106**	**107**	**108**	**111**
* C. lumnitzericola *	MFLUCC 17-0508	T	C	T	T	T	T	C	T	C	G	G	A	C	T	A	T	A	G
* C. thailandica *	MFLUCC 17-0262	T	-	T	-	-	-	T	C	T	C	A	G	-	-	A	C	G	C
* C. thailandica *	MFLUCC 17-0263	T	-	T	-	-	-	T	C	T	C	A	G	-	-	A	C	G	C
* C. xylocarpi *	MFLUCC 17-0251	C	C	C	-	-	C	C	C	C	G	G	G	-	-	G	C	G	G
**Taxon**	**Strain**	**ITS**																	
		**119**	**120**	**121**	**122**	**123**	**124**	**125**	**134**	**157**	**389**	**396**	**404**	**405**	**412**	**413**	**414**	**415**	**420**
* C. lumnitzericola *	MFLUCC 17-0508	T	T	C	-	-	-	-	-	T	T	A	A	-	-	-	-	T	G
* C. thailandica *	MFLUCC 17-0262	C	T	T	C	-	G	G	-	T	T	G	T	T	-	-	-	-	A
* C. thailandica *	MFLUCC 17-0263	C	T	T	C	-	G	G	-	T	T	G	T	T	-	-	-	-	A
* C. xylocarpi *	MFLUCC 17-0251	T	C	T	C	C	G	G	A	G	C	A	A	A	C	T	T	T	G
**Taxon**	**Strain**	**ITS**					**ACT**												
		**439**	**468**	**485**	**487**	**488**	**74**	**78**	**80**	**92**	**95**	**96**	**97**	**107**	**122**	**125**	**129**	**136**	**137**
* C. lumnitzericola *	MFLUCC 17-0508	T	T	C	T	A	G	C	A	T	T	-	-	C	T	A	G	A	A
* C. thailandica *	MFLUCC 17-0262	T	T	T	C	T	T	G	A	A	T	-	-	T	C	T	G	A	G
* C. thailandica *	MFLUCC 17-0263	T	T	T	C	T	T	G	A	A	T	-	-	T	C	T	G	A	G
* C. xylocarpi *	MFLUCC 17-0251	C	C	C	T	T	G	C	T	A	C	C	C	T	C	A	A	G	A
**Taxon**	**Strain**	**ACT**																	
		139	146	147	148	149	150	152	159	165	198	209	210	212	215	216	217	218	223
* C. lumnitzericola *	MFLUCC 17-0508	A	A	G	C	T	C	C	G	T	C	T	C	G	A	A	A	C	A
* C. thailandica *	MFLUCC 17-0262	A	G	-	-	T	T	T	T	T	T	T	C	A	A	A	-	C	A
* C. thailandica *	MFLUCC 17-0263	A	G	-	-	T	T	T	T	T	T	T	C	A	A	A	-	C	A
* C. xylocarpi *	MFLUCC 17-0251	G	G	-	-	A	A	C	T	C	C	A	T	A	T	G	-	A	-
**Taxon**	**Strain**	**ACT**							**RPB2**										
		224	225	231	234	242	245	246	4	18	33	42	57	84	85	96	102	108	120
* C. lumnitzericola *	MFLUCC 17-0508	C	G	C	-	-	A	A	T	T	C	T	C	C	T	T	C	G	A
* C. thailandica *	MFLUCC 17-0262	T	T	C	T	G	T	G	T	C	A	T	C	T	C	T	C	A	G
* C. thailandica *	MFLUCC 17-0263	T	T	C	T	G	T	G	T	C	A	T	C	T	C	T	C	A	G
* C. xylocarpi *	MFLUCC 17-0251	T	T	A	C	G	T	A	C	T	C	C	T	T	C	C	A	A	G
**Taxon**	**Strain**	**RPB2**																	
		**123**	**126**	**129**	**144**	**153**	**171**	**174**	**177**	**204**	**210**	**213**	**216**	**222**	**231**	**237**	**243**	**246**	**279**
* C. lumnitzericola *	MFLUCC 17-0508	C	G	C	G	T	G	C	C	G	C	T	C	T	T	C	T	C	T
* C. thailandica *	MFLUCC 17-0262	T	A	T	A	C	G	T	C	G	T	C	C	C	T	T	T	T	C
* C. thailandica *	MFLUCC 17-0263	T	A	T	A	C	G	T	C	G	T	C	C	C	T	T	T	T	C
* C. xylocarpi *	MFLUCC 17-0251	C	A	C	A	T	A	T	T	C	C	C	T	C	G	C	C	C	T
**Taxon**	**Strain**	**RPB2**																	
		**282**	**294**	**306**	**309**	**336**	**339**	**342**	**351**	**352**	**357**	**378**	**390**	**393**	**396**	**402**	**405**	**435**	**441**
* C. lumnitzericola *	MFLUCC 17-0508	C	A	T	C	T	C	G	T	C	G	A	C	C	G	T	T	C	T
* C. thailandica *	MFLUCC 17-0262	T	G	C	T	C	A	A	C	T	C	G	C	T	A	T	C	C	T
* C. thailandica *	MFLUCC 17-0263	T	G	C	T	C	A	A	C	T	C	G	C	T	A	T	C	C	T
* C. xylocarpi *	MFLUCC 17-0251	T	A	C	C	T	C	G	T	C	C	A	T	T	A	C	T	T	G
**Taxon**	**Strain**	**RPB2**																	
		**456**	**465**	**468**	**492**	**498**	**510**	**516**	**517**	**543**	**561**	**570**	**576**	**603**	**612**	**613**	**615**	**627**	**633**
* C. lumnitzericola *	MFLUCC 17-0508	C	T	C	G	T	T	A	T	T	A	A	G	T	T	C	C	C	G
* C. thailandica *	MFLUCC 17-0262	C	C	G	C	C	C	A	T	C	A	G	A	C	C	T	G	C	G
* C. thailandica *	MFLUCC 17-0263	C	C	G	C	C	C	A	T	C	A	G	A	C	C	T	G	C	G
* C. xylocarpi *	MFLUCC 17-0251	T	T	T	G	T	C	G	C	C	G	G	G	T	C	T	G	G	A
**Taxon**	**Strain**	**RPB2**																	
		**651**	**663**	**675**	**678**	**690**	**693**	**699**	**702**	**711**	**732**								
* C. lumnitzericola *	MFLUCC 17-0508	T	A	C	T	T	G	T	C	C	T								
* C. thailandica *	MFLUCC 17-0262	C	G	T	C	G	A	C	T	C	C								
* C. thailandica *	MFLUCC 17-0263	C	G	T	C	G	A	C	T	C	C								
* C. xylocarpi *	MFLUCC 17-0251	C	A	T	C	T	A	C	C	T	T								

All isolates are new taxa in this study; “-” gap (insertion/deletion); “?” missing data.

## Supplementary Material

XML Treatment for Cytospora lumnitzericola

XML Treatment for Cytospora thailandica

XML Treatment for Cytospora xylocarpi
